# CDX-2 expression correlates with clinical outcomes in MSI-H metastatic colorectal cancer patients receiving immune checkpoint inhibitors

**DOI:** 10.1038/s41598-023-31538-3

**Published:** 2023-03-16

**Authors:** Pina Ziranu, Andrea Pretta, Marta Pozzari, Antonio Maccioni, Manuela Badiali, Daniela Fanni, Eleonora Lai, Clelia Donisi, Mara Persano, Clara Gerosa, Marco Puzzoni, Fabio Bardanzellu, Rossano Ambu, Valeria Pusceddu, Marco Dubois, Giulia Cerrone, Marco Migliari, Sara Murgia, Dario Spanu, Gianluca Pretta, Valentina Aimola, Francesca Balconi, Stefania Murru, Gavino Faa, Mario Scartozzi

**Affiliations:** 1 Medical Oncology Unit, University Hospital and University of Cagliari, SS 554 km 4500 Bivio Per Sestu, 09042 Monserrato, Cagliari, Italy; 2 Genetic and Genomic Laboratory, Pediatric Children Hospital A. Cao ASL8, Cagliari, Italy; 3 Division of Pathology, Department of Medical Sciences and Public Health, University Hospital and University of Cagliari, Cagliari, Italy; 4 Science Department, King’s School Hove, Hangleton Way, Hove, BN3 8BN UK

**Keywords:** Immunotherapy, Tumour immunology

## Abstract

Immune checkpoint inhibitors (ICIs) showed efficacy in metastatic colorectal cancer (mCRC) with mismatch-repair deficiency or high microsatellite instability (dMMR-MSI-H). Unfortunately, a patient’s subgroup did not benefit from immunotherapy. Caudal-related homeobox transcription factor 2 (CDX-2) would seem to influence immunotherapy’s sensitivity, promoting the chemokine (C-X-C motif) ligand 14 (CXCL14) expression. Therefore, we investigated CDX-2 role as a prognostic-predictive marker in patients with mCRC MSI-H. We retrospectively collected data from 14 MSI-H mCRC patients treated with ICIs between 2019 and 2021. The primary endpoint was the 12-month progression-free-survival (PFS) rate. The secondary endpoints were overall survival (OS), PFS, objective response rate (ORR), and disease control rate (DCR). The PFS rate at 12 months was 81% in CDX-2 positive patients vs 0% in CDX-2 negative patients (*p* = 0.0011). The median PFS was not reached (NR) in the CDX-2 positive group versus 2.07 months (95%CI 2.07–10.8) in CDX-2 negative patients (*p* = 0.0011). Median OS was NR in CDX-2-positive patients versus 2.17 months (95% Confidence Interval [CI] 2.17–18.7) in CDX2-negative patients (*p* = 0.026). All CDX-2-positive patients achieved a disease response, one of them a complete response. Among CDX-2-negative patients, one achieved stable disease, while the other progressed rapidly (ORR: 100% vs 0%, *p* = 0.0005; DCR: 100% vs 50%, *p* = 0.02). Twelve patients received 1st-line pembrolizumab (11 CDX-2 positive and 1 CDX-2 negative) not reaching median PFS, while two patients (1 CDX-2 positive and 1 CDX-2 negative) received 3rd-line pembrolizumab reaching a median PFS of 10.8 months (95% CI, 10.8–12.1; *p* = 0.036). Although our study reports results on a small population, the prognostic role of CDX-2 in CRC seems confirmed and could drive a promising predictive role in defining the population more sensitive to immunotherapy treatment. Modulating the CDX-2/CXCL14 axis in CDX-2-negative patients could help overcome primary resistance to immunotherapy.

## Introduction

Nowadays, colorectal cancer (CRC) still represents the third most diagnosed malignancy. Despite recent progress in defining tumor molecular profiles with more personalized therapeutic strategies, CRC remains the second leading cause of cancer-related deaths worldwide^1–4^.

In recent years, the development of immune-checkpoint inhibitors (ICIs) has led clinicians to explore their role in advanced CRC settings^5^. Their efficacy appeared closely related to mutations in mismatch repair (MMR) genes (MSH2, MSH6, MLH1, PMS1, and PMS2), essential for recognizing and repairing DNA errors that occurred during its replication^6–8^. MMR genes inactivation can be due to several mechanisms (e.g., germ-line mutations or point mutations such as insertion and deletions), and this event can be involved in the tumorigenesis process^9^. Microsatellite instability (MSI) results from accumulations of high levels of single-base mismatches or short insertions and deletions in repetitive DNA tracts due to DNA mismatch repair system deficiencies^10^. There are two distinct MSI phenotypes: high-MSI (MSI-H) and low-MSI (MSI-L), distinguished based on the type and number (≥ 40%) of microsatellites analyzed^11^. However, there is no evidence that MSI-L CRC differs in their clinicopathological or molecular features from stable microsatellite tumors (MSS)^11^. About 15% of CRCs are dMMR/MSI-H; these are more likely to have early-stage disease. Advanced d-MMR/MSI-H CRCs are approximately 5%^12,13^.

CRCs with d-MMR/MSI-H are characterized by the production of several cancer neoantigens, increasing tumor mutational burden (TMB) and immune cell infiltration, leading the tumor to be more sensitive to immunotherapy^14^. At the bedside, these observations were confirmed with findings of phase II studies: immune-checkpoint inhibitors administered to pre-treated advanced d-MMR/MSI-H CRCs emerged as a highly effective therapy for these patients^6–8^. On the other hand, in these patients, the 5-Fluorouracil (5-FU) treatment efficacy appears controversial: some authors have described a chemo-resistance associated with a different clinical course and prognosis^15^. In recent phase III Keynote-177 advanced d-MMR/MSI-H CRCs were randomized to receive first-line therapy pembrolizumab vs 5-FU–based therapy with or without bevacizumab or cetuximab, according to molecular cancer signature. The crossover was allowed for patients in the chemotherapy arm following disease progression. Pembrolizumab was demonstrated to be more effective, leading to a meaningful improvement in terms of PFS, response rate (RR), and health-related quality of life, associated with a lower incidence of major treatment-related adverse events^16,17^. In the recently published final survival analysis, the OS benefit from pembrolizumab compared to the control arm was not statistically significant. This result is probably related to the massive crossover (60.4%) of patients in the control arm who received anti-PD1 in the second line^18^. Nevertheless, in the pembrolizumab arm, as shown in the PFS curves, a subgroup of patients did not benefit ab initio from the treatment with the immune checkpoint inhibitor, and the analysis of the radiological response revealed that progressive disease was described as the best response obtained in around 30% of patients enrolled in the experimental arm^16^. Therefore, immunotherapy would appear ineffective in a subset of patients with advanced d-MMR/MSI-H CRC.

Thus, biomarkers need to be further investigated as predictive factors to guide clinicians' treatment strategies. CDX2 is a nuclear homeobox transcription factor that belongs to the caudal-related family of CDX homeobox genes. CDX-2 is crucial for axial patterning of the alimentary tract during embryonic development, and it is involved in cell proliferation, differentiation, adhesion, and apoptosis processes^19^. In adults, CDX-2 is only expressed by intestine cells and can be used to identify the colorectal origin of adenocarcinomas^20^. This gene can be downregulated by oncogenic pathways in CRC^21^.

Over the last decades, the loss of the CDX-2 expression has been associated with disease progression, vessel invasion, and metastasis, and CDX-2 negative CRCs are associated with poor prognosis and aggressive clinical behavior^21,22^. Dalerba et al. showed that lack of CDX-2 expression is correlated with high-risk stage II colon cancer that may probably benefit from adjuvant chemotherapy^23^, and Aasebo et al. found that CDX-2 immunochemistry loss is an independent negative prognostic factor for survival in mCRC^24^. In contrast, CDX-2 expression defines a group of BRAF mutated cases with better prognoses^24^.

However, the relationship between CDX-2 expression and immunotherapy response remains unclarified. In a recent study, Wang et al. described that CDX-2 promotes the CXCL14 expression by activating its enhancer, promoting natural killer cell-mediated immunotherapy^25^. Natural killer (NK) cells, known for their innate and cytotoxic characteristics, were initially identified as a population of spleen-derived cytotoxic lymphocytes that were neither B- nor T-lymphocytes^26^. NK cells act as the first line of defence against pathogens and tumours through their effects on death receptor pathways and granule exocytosis, analogous to those of cytotoxic T lymphocytes (CTLs)^27^. CXCL14, also known as BRAK and expressed in a wide range of normal cells, is particularly abundant in epithelial cells. CXCL14 is also expressed in immune cells (such as monocyte, B cells, THP-1 cell line and monocyte-derived dendritic cells), which are involved in immune surveillance through the recruitment of NK, dendritic, and T cells^28,29^ .Therefore, overexpression of CXCL14 would increase the migration and cytotoxicity of NK cells. In Wang's study, CDX2 could regulate CXCL14 expression by activating its enhancer. CDX2 can therefore increase the migration and cytolytic activity of NK cells by upregulating CXCL14, and the CDX2/CXCL14 axis inhibits tumorigenesis through NK cells^25^. NK cells can induce targeted cytolysis by producing IFN-γ and TNF-α release^25^. Thus, CDX2 might induce the migration and infiltration of NK cells, increases the secretion of IFN-γ and TNF-α by NK cells, enhances NK cell toxicity against cancer cells, and suppresses tumour growth^25^.

Currently, the role of CDX-2 expression in advanced d-MMR/MSI-H CRC patients subjected to immune checkpoint inhibitors has not been further investigated. Based on these considerations, we have conducted an analysis of CDX-2 expression in patients with mCRC treated with ICIs to investigate the role of CDX-2 as a prognostic-predictive marker in this group of patients.

## Materials and methods

### Study design

We retrospectively collected data from MSI-H mCRC patients treated with pembrolizumab between 2019 and 2021 at the Medical Oncology Unit of the University Hospital of Cagliari. Pembrolizumab was administered at a dose of 200 mg every 3 weeks. Baseline demographic and clinical characteristics, treatment and survival information were collected from clinical charts. Pathological and molecular features were retrieved from histological reports. The following data were collected: gender, age, and Eastern Cooperative Oncology Group (ECOG) Performance Status (PS) at diagnosis of metastatic disease, the onset of metastatic disease, primary tumor location, sites of metastases, mucinous histology, grade of differentiation, CDX2 tumor expression, BRAF/RAS mutational status, MSI/MMR status, and treatment outcome. For study purposes, right-sided and left-sided CRC primary tumors were defined as proximal or distal to the splenic flexure.

Approval for the study was obtained by Ethics Committee of the University Hospital of Cagliari (Protocol number 2020/10,912—code: EMIBIOCCOR) and written informed consent was obtained from all participants for their tissues to be utilized for this work.

The aim of the present analysis was to evaluate the role of CDX2 tumor expression in predicting the clinical outcome in MSI-H mCRC patients treated with pembrolizumab. The primary endpoint was the PFS-rate at 12 months. The secondary endpoints were OS, PFS, ORR, and DCR.

### Histological, immunohistochemical and molecular analysis

Tumor samples were routinely processed for histological observation and stained with hematoxylin–eosin (H.E). For immunohistochemical (IHC) analysis, 3 µm thick sections were obtained from the paraffin block. All reagents were purchased from Ventana Medical Systems Inc. 1910 E. Innovation Park Drive Tucson, Arizona 85,755 USA. The sections were automatically dewaxed and rehydrated with EZ Prep 1X (Ref. 950–102) and pre-treated with heat-induced epitope retrieval in Ultra CC1 (Ref. 950–224), following Dealer’s instructions. Slides were then incubated at room temperature with anti-human CDX2 Rabbit monoclonal antibody – clone EPR2764Y – (Ref. 760–4380). All immunostaining procedures were performed using the UltraView Universal DAB Detection Kit (Ref. 760–5000) on the BenchMark Ultra (Ventana Medical Systems Inc. 1910 E. Innovation Park Drive Tucson, Arizona 85,755 USA) instrument, according to the manufacturer’s instructions^30^. RAS and BRAF gene mutational status was assessed by pyrosequencing on formalin-fixed, paraffin-embedded (FFPE) archival tumor tissue samples from primary tumors or metastases. Expression of MMR proteins (MLH1, MSH2, MSH6, and PMS2) was performed by immunohistochemistry. Histological, immunohistochemical and molecular analysis were conducted at the Division of Pathology of University Hospital of Cagliari.

MSI analysis was also carried out on all samples. DNAs from FFPE tumor samples and from peripheral blood of the same patient were analyzed by the TITANO MSI kit (Diatech Pharmacogenetics, Jesi, Italy) following the manufacturer’s protocol, using mono-bi and tetranucleotide repeats. These include target markers (BAT-25, BAT-26, D2S123, D17S250, D5S346, BAT40, NR-21, NR-24, D18S58 and TGFBRII) and two control markers for the detection of possible contamination or sample mixups (TPOX and TH01). The procedure consists of multiplex PCR amplifications with fluorescent primers and subsequent DNA fragment analysis on an automated sequencer (ABI PRISM 3130XL Genetic Analyzer—Applied Biosystem).

A diagnosis of microsatellite stability (MSS) can be made if no unstable microsatellite is found at any locus by comparing tumor and normal tissue. Conversely, if a different number of short-repeated DNA sequences is detected between the two tissues, we can diagnose the MSI status and classify it as low (MSL-L) if the number of instabilities is between 1 and 3, or high (MSI-H) if the number is ≥ 4^12,31,32^.

MSI analysis was performed at Genetic and Genomic Laboratory of Microcitemico Children's Hospital A. Cao of Cagliari.

### Statistical analysis

Statistical analysis was performed with the MedCalc Statistical Software Version 14.10.2 (MedCalc Software bvba, Ostend, Belgium; http://www.medcalc.org; 2014). The association between categorical variables was estimated by the Fisher exact test for categorical binomial variables or by the chi-square test in all other instances. Survival probability over time was estimated by the Kaplan–Meier method. Significant differences in the probability of survival between the strata were evaluated by the log-rank test. The independent role of variables that were statistically significant at a univariate analysis was assessed with a logistic regression analysis. OS was defined as the time interval between the date of the beginning of pembrolizumab treatment to death or the last follow-up visit for patients who were lost at follow-up. PFS was defined as the interval between the date of the beginning of pembrolizumab treatment to death, first sign of clinical progression or the last follow-up visit for patients who were lost at follow-up. ORR was defined as the percentage of patients who achieved a partial or complete response to treatment according to RECIST Version 1.1. DCR was defined as the percentage of patients with stable disease or partial/complete response to treatment.

In order to detect a difference in the effect size with statistical significance in the proportion of patients without disease progression at 12 months according to CDX-2 status and assuming a 12 months PFS of 55% in the CDX2 expressed population and 0% in patients with loss of CDX-2 expression, at least 12 patients were necessary with α = 0.2 and β = 0.2, using a “comparison of proportion test.” A *p*-value < 0.05 was considered statistically significant.

### Ethical approval

This study was performed in accordance with the study protocol, the ethical principles stated in the Declaration of Helsinki as well as those indicated in the International Conference on Harmonization (ICH) Note for Guidance on Good Clinical Practice (GCP; ICH E6, 1995), and all applicable regulatory requirements.

### Consent to participate

All patients signed a written informed consent before study entry. Adequate information was given to eligible patients by the principal investigator or co-investigators in accordance with local regulations. The declaration of informed consent was personally signed and dated by the subject, and by the investigator/person designated by the investigator to conduct the informed consent discussion.

## Results

### Patients characteristics

Of the 300 patients analyzed, 14 consecutive mCRC patients with MSI-H profiles were treated with pembrolizumab at the Medical Oncology Unit of Cagliari University Hospital from 2019 to 2021. Two mCRC patients with MSI-L profile were also detected and excluded from our study, both CDX2 positive.

The patient characteristics of our study population were consistent with a stage IV MSI-H CRC population (Table 1). The median age was 63 years (range, 55–72). Of the 14 patients 2 (14.3%) had negative CDX-2 expression, the remaining 12 (85.7%) had CDX-2 expressed. At diagnosis, patients with metastatic disease were 10 (83.3%) in the CDX-2 positive subgroup and one (50%) in the CDX-2 negative subgroup. All patients received pembrolizumab; 11 (91.7%) CDX-2 positive patients and one (50%) CDX-2 negative patient in first line while one (8.3%) CDX-2 positive patient and one (50%) CDX-2 negative patient in third line. Twelve patients were RAS WT (10 CDX2 positive and 2 CDX2 negative) and 2 RAS mutated (both CDX2 positive). Of the 14 patients, 2 were BRAF V600E mutated, both CDX2 positive. As of the data cut-off date, February 01, 2023, one CDX2-positive patient (8.3%) died while the other 11 (91.7%) were alive. Of these eleven CDX2-positive patients alive, 8 (66.7%) continued pembrolizumab, 2 (16.7%) had disease progression and continued second-line 5-fluorouracil-based chemotherapy. In contrast, the 2 CDX-2 negative patients were dead. Both patients who received III-line pembrolizumab had metastatic right colon cancer with a RAS and BRAF WT profile. They then underwent first-line treatment with 5-fluorouracil-based doublet (mFOLFOX) plus anti-VEGF and in II-line doublet with 5-fluorouracil-based doublet (FOLFIRI) plus anti-VEGF beyond progression. Both patients achieved modest benefits from I- and II-line treatment. The time from the start of I-line to the start of pembrolizumab was 10 months in the CDX-2-positive patient and 10,5 months in the CDX-2-negative patient. Treatment with pembrolizumab as monotherapy was well tolerated in the I and III lines. No hematologic toxicity was reported. Immune-mediated adverse events occurred in 5 patients (35%). 2 patients presented grade 2 hypothyroidism, treated with adjustment of hormone therapy through multidisciplinary management with endocrinologists. Three patients presented skin reaction: 1 patient had grade 1 pruritus, and the other two had a grade 2 maculopapular rush, treated with antihistamines and topical steroids with benefit, as directed by the dermatologist. There were no colitis or hepatitis.

**Table 1:** Patients’ characteristics.

Characteristics	CDX-2-positive n (%)	CDX-2-negative n (%)
N. of patients	12	2
Gender		
Male	8 (66.7%)	2 (100%)
Female	4 (33.3%)	–
Age		
< 65	6 (50%)	1 (50%)
≥ 65	6 (50%)	1 (50%)
ECOG PS		
0	4 (33.3%)	–
1	8 (66.7%)	2 (100%)
Site of primary tumor		
Left-sided colon	3 (25%)	–
Right-sided colon	9 (75%)	2 (100%)
Metastases		
Single site	6 (50%)	1 (50%)
Multiple site	6 (50%)	1 (50%)
Metastatic disease		
Synchronous	10 (83.3%)	1 (50%)
Metachronous	2 (16.7%)	1 (50%)
Liver metastases		
Yes	8 (66.7%)	–
No	4 (33.3%)	2 (100%)
Peritoneal metastases		
Yes	4 (33.3%)	1 (50%)
No	8 (66.7%)	1 (50%)
Tumor grade differentiation		
Well-moderate	3 (41.7%)	–
Poorly	7 (58.3%)	2 (100%)
Angioinvasion		
Yes	10 (83.3%)	2 (100%)
No	2 (16.7%)	–
*K-RAS/N-RAS* mutational status		
Wild type	10 (83.3%)	2 (100%)
Mutant	2 (16.7%)	–
*B-RAF* mutational status		
Wild type	10 (83.3%)	2 (100%)
Mutant	2 (16.7%)	–
Pembrolizumab treatment line		
I	11 (91.7%)	1 (50%)
III	1 (8.3%)	1 (50%)

ECOG PS: Eastern Cooperative Oncology Group Performance Status; K-RAS: Kirsten rat sarcoma virus; N-RAS: neuroblastoma ras viral oncogene homolog; B-RAF: v-Raf murine sarcoma viral oncogene homolog B.

### Treatment outcomes

**Figure 1:**
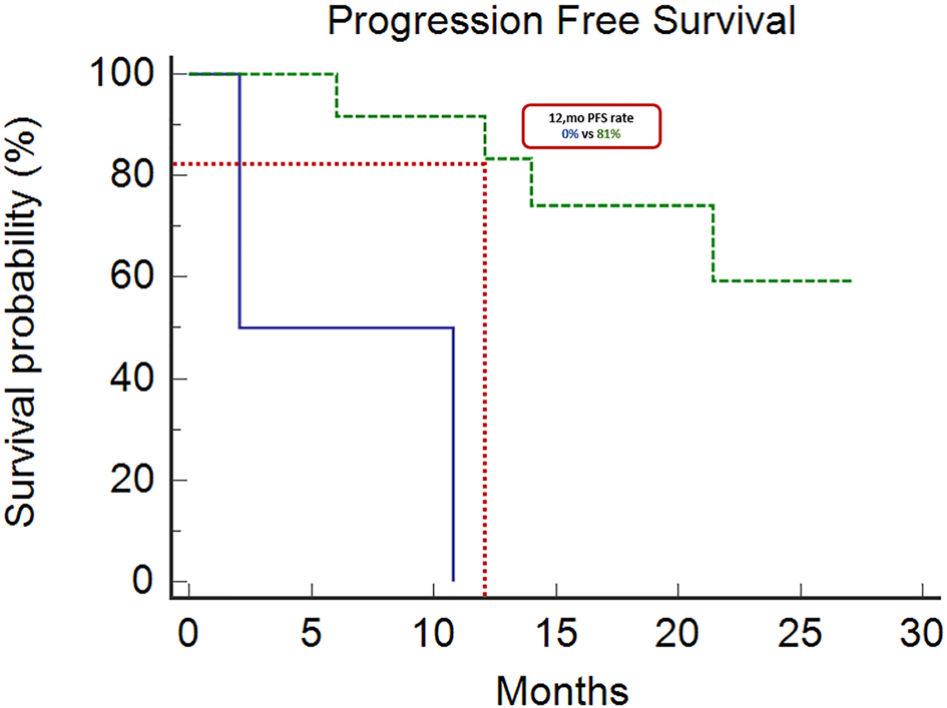
Progression Free Survival: The median PFS was not reached in CDX-2-positive patients (dotted/green line) versus 2.07 months (95%CI 2.07–10.8) in CDX2-negative patients (continuous/blue line) (*p* = 0.0011) The proportion of patients without disease progression at 12 months was 81% in the CDX-2-positive group. All the two CDX-2-negative patients had discontinued treatment due to disease progression.

**Figure 2:**
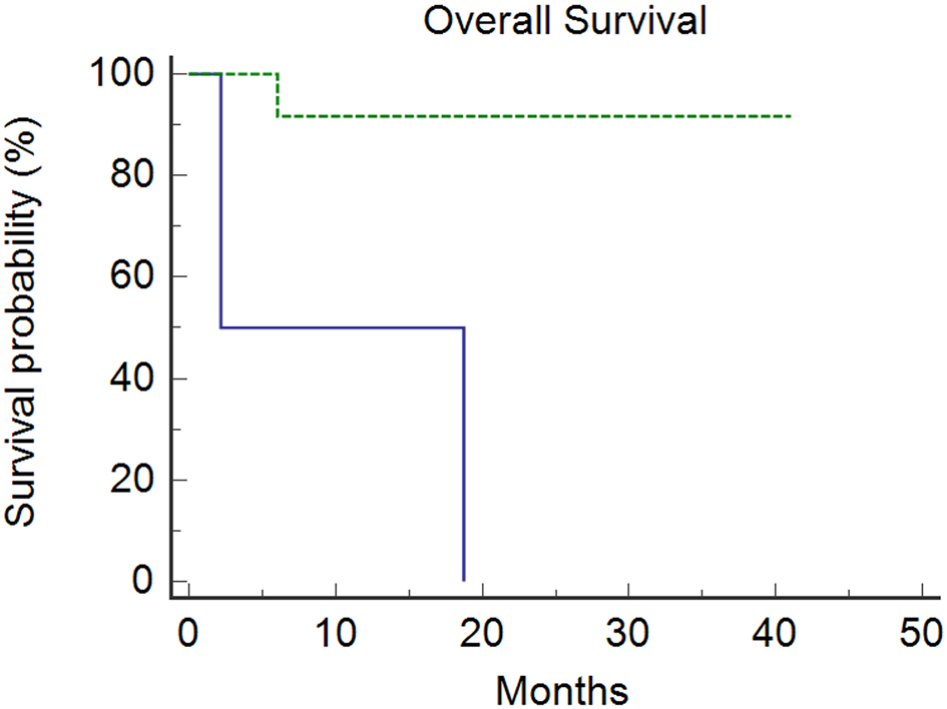
Overall Survival: Median Overall Survival was not reached in CDX-2-positive patients (dotted/green line) versus 2.17 months (95%CI 2.17–18.7) in CDX-2-negative patients (continuous/blue line) (*p* = 0.026).

At a median follow-up of 19.8 months (95%CI 14.1–23.2), the median OS was not reached. The proportion of patients without disease progression at 12 months was 81% in the CDX-2-positive group. All the two CDX-2-negative patients had discontinued treatment due to disease progression (Fig. 1). The median PFS was not reached in CDX-2-positive patients versus 2.07 months (95%CI 2.07–10.8) in CDX2-negative patients (*p* = 0.0011) (Fig. 1). Median OS were not reached in CDX-2-positive patients versus 2.17 months (95%CI 2.17–18.7) in CDX-2-negative patients (*p* = 0.0262) (Fig. 2).

All CDX-2-positive patients achieved a disease response, one of them a complete response. One achieved stable disease among the CDX-2-negative patients, while the other progressed rapidly (ORR: 100% vs 0%, *p* = 0.0005; DCR: 100% vs 50%, *p* = 0.02) (Table 2).

**Table 2:** Response rate.

Response	CDX-2-positive	CDX-2-negative
Best response, n (%)		
Complete response	1 (8.3%)	–
Partial response	11 (91.7%)	–
Stable disease	–	1 (50%)
Progression disease	–	1 (50%)
ORR, n (%)	12 (100%)	–
DCR, n (%)	12 (100%)	1 (50%)

ORR: overall response rate; DCR: disease control rate.

We analyzed the impact of different clinicopathological features on OS and PFS. At the univariate analysis, the only variable that significantly correlated with median PFS was the line of pembrolizumab administration. Pembrolizumab in the first line did not reach the median PFS, versus the same anti-PD-1 achieved a median PFS of 10.8 months (95%CI 10.8–12.1; *p* = 0.036) in the third line. Other variable evaluated did not show a significant correlation with OS or PFS (Table 3).

**Table 3:** Variable correlation with overall survival and progression free survival at univariate analysis.

Variable (N)	PFS—*P* value	OS—*P* value
Gender		
M (10)	0.07	0.4
F (4)		
Age		
≥ 65 years (7)	0.59	0.34
< 65 years (7)		
ECOG PS		
0 (4) 1 (10)	0.23	0.23
Site of primary tumor		
Left-sided colon (3)	0.63	0.34
Right-sided colon (9)		
Primary tumor resection		
Yes (6)	0.7	0.8
No (8)		
Tumor grade		
Well-moderate (7)	0.73	0.052
Poorly (7)		
Angioinvasion		
No (2)	0.9	0.4
Yes (12)		
Metastases		
Single site (7)	0.37	0.69
Multiple sites (7)		
Metastatic disease		
Synchronous (11)	0.17	0.6
Metachronous (3)		
Liver metastases		
No (6)	0.78	0.33
Yes (8)		
Peritoneal metastases		
No (9)	0.13	0.66
Yes (5)		
K-RAS/N-RAS mutational status		
RAS WT (12)	0.25	0.48
RAS MUT (2)		
B-RAF mutational status		
B-RAF WT: (12)	0.28	0.48
B-RAF MUT (2)		
Pembrolizumab treatment line		
I line (12)	**0.036**	0.49
III line (2)		
NLR		
≤ 2.7 (7)	0.24	0.07
< 2.07 (7)		
Hemoglobin		
≤ 10.9 g/dl (5)	0.28	0.24
> 10.9 gr/dl (9)		
CEA		
Normal CEA (5)	0.19	0.14
High CEA (9)		
LDH		
Normal LDH (8)	0.57	0.42
High LDH (6)		

N: number of patients; PFS: Progression Free Survival; OS: Overall Survival; K-RAS: Kirsten rat sarcoma virus; N-RAS: neuroblastoma ras viral oncogene homolog; B-RAF: v-Raf murine sarcoma viral oncogene homolog B; ECOG PS: Eastern Cooperative Oncology Group Performance Status; WT: wild type, MUT: mutated; NLR: Neutrophil to Lymphocyte Ratio; LDH: lactate dehydrogenase.

## Discussion

The emergence of ICIs, mainly including anti-programmed cell death protein 1/programmed cell death ligand 1 (PD-1/PD-L1) and anti-cytotoxic T lymphocyte-associated antigen-4 (CTLA-4) monoclonal antibodies, have dramatically changed the therapeutic landscape over the last decade. A durable clinical response is achieved by disrupting immune tolerance and activating cytotoxic T-cells in refractory patients with solid tumors, including a small subset of patients with mCRC^6–9,33–37^. ICIs demonstrated promising efficacy in dMMR/MSI-H mCRC, providing clinical benefits superior to standard treatments and leading to the Food and Drug Administration (FDA) and European Medicines Agency (EMA) approval of anti-PD-1 in this setting^38–40^.

Initial data on the ICIs activity in MSI-H CRC were observed in a phase I study evaluating an anti-PD-1 in the treatment of refractory solid tumors. In this trial, one MSI-H CRC patient obtains a 3-year long complete response, underlying the potential of ICIs in this CRC subgroup^41^. Subsequent phase II studies (KEYNOTE-016, KEYNOTE-164, KEYNOTE-158, KEYNOTE-012, and KEYNOTE-028) confirmed the efficacy of pembrolizumab (anti-PD-1) in the treatment of refractory MSI-H mCRC. In total, 90 patients were evaluated. The ORR was 39.6% (95% CI, 31.7–47.9) and lasted more than six months in 78% of patients. These results led to the FDA's fast-track approval of pembrolizumab for chemo-refractory MSI-H mCRC in 2017^7,42^. The phase II, non-randomized, multi-study CHECKMATE-142 study suggested that the combination of ipilimumab with nivolumab might be superior to nivolumab monotherapy for the treatment of chemo-refractory mCRC MSI-H (ORR 54.6% vs 31%; DCR 80% vs 69%, respectively)^44^. This data led the FDA to approve nivolumab, with or without ipilimumab, to treat previously treated CRC MSI-H. In the first-line cohort of the CHECKMATE-142 trial, nivolumab combined with ipilimumab achieved an ORR of 69% (95% CI, 53–82%) and a DCR of 84% (95% CI, 70.5–93.5%). Median OS was not reached, and 79% were alive at 24 months (95% CI, 64.1–88.7%)^45,46^. Recently updated data from the randomized phase III KEYNOTE-177 trial confirmed the efficacy of pembrolizumab compared to Standard of Care (SOC) in treatment-naïve MSI-H mCRC patients. At final analysis, median PFS was 16.5 months (95% CI: 5.4–32.4), and median OS was not achieved (NR; 95% CI 49–2-NR) in pembrolizumab arm versus median PFS of 8.2 months (95% CI: 6.1–10.2) and median OS of 36.7 months (27.6-NR) in SOC arm, leading to FDA approval of pembrolizumab in June 2020 for the first-line treatment of metastatic or unresectable MSI-H CRC^18^.

Therefore, immunotherapy has been shown to be effective in this setting. The microsatellite instability/mismatch repair proteins deficit is an excellent biomarker to distinguish immunotherapy-benefited populations, but it is probably insufficient. In fact, in the first line setting trial, the Authors observed that 13% of patients were refractory to the nivolumab/ipilimumab combination in CHECKMATE 142^47^, while 29.4% of patients in the KEYNOTE-177 trial were refractory to pembrolizumab^16^.

Consequently, there has been an increased focus on searching for potential biomarkers in the ICIs effectiveness. In the tumor-agnostic trial of Le et al., PD-L1 expression was not correlated with the benefit of immunotherapy in patients with dMMR/MSI-H mCRC^6,44,46^. BRAF V600E, present in approximately 30–40% of dMMR/MSI-H mCRC, was also not associated with the efficacy of checkpoint inhibitors (6.41). There are contradictory data about the possible role of the RAS mutation that seems to reduce the efficacy of ICIs in the KEYNOTE 177 study, whereas, in the CHECKMATE 142 study, the benefit of ICIs seems to be preserved regardless of RAS status^8,47,48^. Peritoneal metastasis with ascites appears to be associated with ICI resistance^49^.

Recent studies have suggested several potential biomarkers that could identify the immunotherapy-resistant population among dMMR/MSI-H patients, such as TMB^50–52^, polymerase epsilon (POLE) mutation^53,54^, gene fusions such as NTRK, BRAF, RET, FGFR, ROS1 and ALK^55,56^, *Fusobacterium nucleatum*-enriched microbiota^57,58^, interferon γ pathway and β2-microglobulin mutations, and elevated expression of vascular endothelial growth factor A (VEGF-A)^59,60^, with exciting but still inconclusive results.

**Figure 3:**
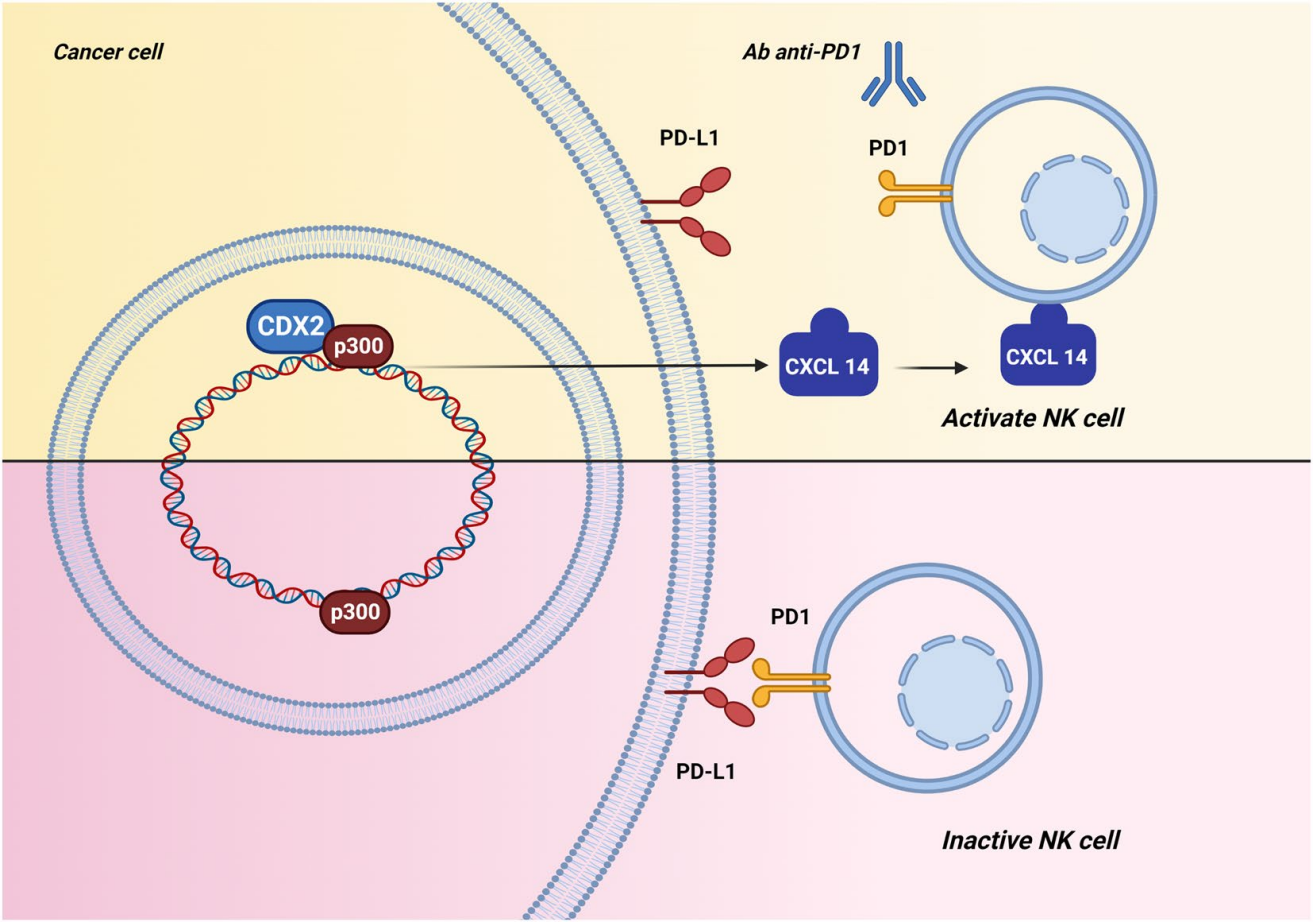
The relationship between CDX-2 expression and immunotherapy response: CDX-2 interacts with histone acetyltransferase p300. The CDX-2/p300 complex activates the enhancer of CXCL14 to promote its expression. Lack of expression of the CDX2/CXCL14 axis reduces the recruitment of immune cells to the microenvironment, inhibiting the therapeutic efficacy of natural killer cell-mediated cancer immunotherapy.

Recent pre-clinical research investigated the underlying molecular mechanism whereby CXCL14, mediates natural killer cells to target head and neck squamous cell carcinoma^25^. Wang H et al. found that CDX-2 activates the CXCL14 enhancer to up-regulate its expression and may improve the therapeutic efficacy of immunotherapy against cancer by natural killer cells^25^. CDX-2 interacts with histone acetyltransferase p300, and consequently, CDX-2/p300 activates the enhancer of CXCL14 to promote its expression^25^. CXCL14 is expressed in normal cells, especially abundant in epithelial cells and immune cells, where it is involved in immune surveillance by recruiting natural killer cells, dendritic cells, and T cells in microenvironment^61,62^. As mentioned, NK cells are innate lymphocytes with cytotoxic activity against tumor cells mediated by the release of cytokines and chemokines^63^. Unlike T cells, NK can recognize tumor cells without overexpression of neoantigens or autoantigens. They can recognize tumor-associated surface proteins and changes in surface expression of major histocompatibility complex class 1 (MHC-1) molecules that often characterize the malignant transformation of cancer^64^. It has been reported that PD-1 is expressed in T cells, B cells, and NK cells, playing an essential role in regulating the threshold, strength, duration, and properties of antigen-specific immune responses^65^. High expression of inhibitory molecules, such as PD-1, leads to dysfunction and apoptosis of NK cells, reducing antitumor activity^66^. In addition, several studies have reported that PD-L1 expression in tumor cells results in reduced NK cell responses. Therefore, PD-1/PD-L1 blockade mediated by the checkpoint inhibitors elicits an antitumor response from NK cells^67–69^. It is reasonable to propose that, in addition to T cells, NK cells participate in the clinical benefit of anti–PD-1/PD-L1 antibody therapy by directly killing tumor cells and/or recruiting T cells^67^. A lack of the CDX2/CXCL14 axis expression would reduce the recruitment of immune cells in the microenvironment, inhibiting the natural killer cell-mediated immunotherapy and reducing the activity of immune checkpoint inhibitors, such as pembrolizumab (Fig. 3).

The prognostic role of CDX-2 and CXCL14 is known as reported in several studies^21–23,70,71^. Based on this evidence, we conducted our analysis to evaluate a possible predictive role of immunotherapy primary resistance in MSI-H/dMMR mCRC patients with CDX-2 negative expression. Although the small numbers, the findings of our study showed a clear difference in terms of OS, TTP, and ORR between positive and negative CDX2 patients. Advanced CRC patients with CDX-2 negative expression achieved a rapid disease progression on pembrolizumab therapy. The results of our analysis seem to confirm the prognostic role of CDX-2 in CRC. Furthermore, the profound difference in PFS (Fig. 1) and RR (Table 2) suggested an auspicious role in defining the population that could benefit from immunotherapy.

At univariate analysis, no other variable had such a marked impact on efficacy outcomes. In particular, the mutational profile of BRAF did not impact the response to anti-PDL-1, in line with the Literature data. RAS mutational status and peritoneal carcinosis reduced the immunotherapy efficacy, but the data was not statistically significant. Regarding hematologic analysis, high baseline CEA values, high Neutrophil to Lymphocyte Ratio (NLR) values, and high **lactate dehydrogenase.**

(LDH) values seem associated with a worse prognosis, in line with literature data^72–74^ but the trend is not statistically significant. In contrast, primary tumor resection, negative primary tumor lymph nodes, low differentiation grading, absence of angioinvasion, oligometastatic disease, and a left-sided primary tumor seem correlated with a better prognosis, in line with the literature data^75–77^ but the finding is not statistically significant. The small sample size and population selected for MSI-h, which tended to have a good prognosis, undoubtedly influenced the results of our analysis.

The only other variable that impacted on PFS data is the line of pembrolizumab treatment. Patients with MSI-H would benefit less from 5-fluorouracil therapies^15^, so using a checkpoint inhibitor at an earlier treatment line might impact the outcomes of patients. In our analysis, this finding is confirmed in terms of PFS. Patients treated in the first line with pembrolizumab had a better benefit than patients treated in line III (*p* = 0.036). This data was not confirmed in terms of OS and RR.

More extensive studies and longer follow-up are needed to draw conclusions on the impact of different clinicopathological features in ICI-treated patients. Furthermore, prospective studies will help confirm the role of CDX-2, but such a marked effect in defining an immunotherapy-resistant population holds excellent promise. Future studies will further investigate this topic by evaluating tumor-infiltrating lymphocytes and CXCL14 expression in the tumor microenvironment, especially comparing CDX-2 positive cases with CDX-2 negative cases. It will also be interesting to evaluate PD-L1 expression in CDX2-positive and -negative patients and see if the lack of correlation with response to immunotherapy in mCRC, as described by other works^6,44,46,78^, is confirmed.

Of considerable importance is that CDX-2 could be an excellent predictive marker of ICIs poor efficacy due to the ease and low cost of the immunohistochemical analysis. Finally, a targeted therapy that can activate the CDX-2/CXCL14 axis even in CDX-2 negative patients could represent an important milestone in overcoming primary resistance to immunotherapy.

Therefore, the potential predictive role, the excellent cost-effectiveness, and possible modulation of ICIs response by acting on CDX-2 could bring this old marker to a promising increased application in daily clinical practice.

## Data Availability

Datasets generated during and/or analyzed during the current study are available from the corresponding author on reasonable request.

## Abbreviations

CDX-2: Caudal-related homeobox transcription factor 2
CRC: Colorectal cancer
DCR: Disease control rate
dMMR: Mismatch-repair-deficiency
ICI: Immune-checkpoint inhibitors
mCRC: Metastatic colorectal cancer
MMR: Mismatch repair
MSI: Microsatellite instability (H: high; L: low)
MSS: Microsatellite stability
ORR: Objective response rate
OS: Overall survival
PFS: Progression free survival
TMB: Tumour mutation burden

## References

[1] Sung H, Ferlay J, Siegel RL (2021). Global cancer statistics 2020: GLOBOCAN estimates of incidence and mortality worldwide for 36 cancers in 185 countries. CA Cancer J. Clin..

[2] Lai E, Liscia N, Donisi C (2020). Molecular-biology-driven treatment for metastatic colorectal cancer. Cancers.

[3] Puzzoni M, Ziranu P, Demurtas L (2020). Why precision medicine should be applied across the continuum of care for metastatic colorectal cancer patients. Future Oncol. Lond. Engl..

[4] Ziranu P, Lai E, Schirripa M (2021). The role of p53 expression in patients with RAS/BRAF wild-type metastatic colorectal cancer receiving irinotecan and cetuximab as later line treatment. Target Oncol..

[5] Basile D, Garattini SK, Bonotto M (2017). Immunotherapy for colorectal cancer: Where are we heading?. Expert Opin. Biol. Ther..

[6] Le DT, Uram JN, Wang H (2015). PD-1 blockade in tumors with mismatch-repair deficiency. N. Engl. J. Med..

[7] Le DT, Kim TW, Van Cutsem E (2020). Phase II open-label study of pembrolizumab in treatment-refractory, microsatellite instability-high/mismatch repair-deficient metastatic colorectal cancer: KEYNOTE-164. J. Clin. Oncol. Off. J. Am. Soc. Clin. Oncol..

[8] Overman MJ, McDermott R, Leach JL (2017). Nivolumab in patients with metastatic DNA mismatch repair-deficient or microsatellite instability-high colorectal cancer (CheckMate 142): An open-label, multicentre, phase 2 study. Lancet Oncol..

[9] Goel A, Arnold CN, Boland CR (2001). Multistep progression of colorectal cancer in the setting of microsatellite instability: New details and novel insights. Gastroenterology.

[10] Koopman M, Kortman GAM, Mekenkamp L (2009). Deficient mismatch repair system in patients with sporadic advanced colorectal cancer. Br. J. Cancer..

[11] Pawlik TM, Raut CP, Rodriguez-Bigas MA (2004). Colorectal carcinogenesis: MSI-H versus MSI-L. Dis. Mark..

[12] Boukouris AE, Theochari M, Stefanou D (2022). Latest evidence on immune checkpoint inhibitors in metastatic colorectal cancer: A 2022 update. Crit. Rev. Oncol. Hematol..

[13] Ganesh K, Stadler ZK, Cercek A (2019). Immunotherapy in colorectal cancer: Rationale, challenges and potential. Nat. Rev. Gastroenterol. Hepatol..

[14] DeAngelis GL, Bottarelli L, Azzoni C (2018). Microsatellite instability in colorectal cancer. Acta Bio.-Med. Atenei Parm..

[15] Tougeron D, Sueur B, Zaanan A (2020). Prognosis and chemosensitivity of deficient MMR phenotype in patients with metastatic colorectal cancer: An AGEO retrospective multicenter study. Int. J. Cancer..

[16] André T, Shiu KK, Kim TW (2020). Pembrolizumab in microsatellite-instability-high advanced colorectal cancer. N. Engl. J. Med..

[17] Andre T, Amonkar M, Norquist JM (2021). Health-related quality of life in patients with microsatellite instability-high or mismatch repair deficient metastatic colorectal cancer treated with first-line pembrolizumab versus chemotherapy (KEYNOTE-177): An open-label, randomised, phase 3 trial. Lancet Oncol..

[18] Diaz LAJ, Shiu KK, Kim TW (2022). Pembrolizumab versus chemotherapy for microsatellite instability-high or mismatch repair-deficient metastatic colorectal cancer (KEYNOTE-177): Final analysis of a randomised, open-label, phase 3 study. Lancet Oncol..

[19] Saad RS, Ghorab Z, Khalifa MA, Xu M (2011). CDX2 as a marker for intestinal differentiation: Its utility and limitations. World J. Gastrointest. Surg..

[20] Kaimaktchiev V, Terracciano L, Tornillo L (2004). The homeobox intestinal differentiation factor CDX2 is selectively expressed in gastrointestinal adenocarcinomas. Mod. Pathol. Off. J U. S. Can. Acad. Pathol. Inc..

[21] Graule J, Uth K, Fischer E (2018). CDX2 in colorectal cancer is an independent prognostic factor and regulated by promoter methylation and histone deacetylation in tumors of the serrated pathway. Clin Epigenet..

[22] Bae JM, Lee TH, Cho NY, Kim TY, Kang GH (2015). Loss of CDX2 expression is associated with poor prognosis in colorectal cancer patients. World J. Gastroenterol..

[23] Dalerba P, Sahoo D, Paik S (2016). CDX2 as a prognostic biomarker in stage II and stage III colon cancer. N. Engl. J. Med..

[24] Aasebø K, Dragomir A, Sundström M (2020). CDX2: A prognostic marker in metastatic colorectal cancer defining a better BRAF mutated and a worse KRAS mutated subgroup. Front Oncol..

[25] Wang H, Nan S, Wang Y, Xu C (2021). CDX2 enhances natural killer cell-mediated immunotherapy against head and neck squamous cell carcinoma through up-regulating CXCL14. J. Cell Mol. Med..

[26] Guillerey C, Huntington ND, Smyth MJ (2016). Targeting natural killer cells in cancer immunotherapy. Nat. Immunol..

[27] Sharma P, Kumar P, Sharma R (2017). Natural killer cells—their role in tumour immunosurveillance. J. Clin. Diagn. Res..

[28] Lu J, Chatterjee M, Schmid H (2016). CXCL14 as an emerging immune and inflammatory modulator. J. Inflamm. (Lond.)..

[29] Westrich JA, Vermeer DW, Silva A (2019). CXCL14 suppresses human papillomavirus-associated head and neck cancer through antigenspecific CD8(+) T-cell responses by upregulating MHC-I expression. Oncogene.

[30] Aimola V, Fanni D, Gerosa CG (2022). Balance between the stem cell marker CD44 and CDX2 expression in colorectal cancer. Ann. Res. Oncol..

[31] Malapelle U, Parente P, Pepe F (2021). Evaluation of micro satellite instability and mismatch repair status in different solid tumors: A multicenter analysis in a real world setting. Cells.

[32] Boland CR (2007). Clinical uses of microsatellite instability testing in colorectal cancer: An ongoig challenge. J. Clin. Oncol..

[33] Dubois M, Liscia N, Brunetti O (2022). The role of immune checkpoint inhibitors in the treatment sequence of advanced gastric or gastro-esophageal junction cancer: A systematic review and meta-analysis of randomized trials. Crit. Rev. Oncol. Hematol..

[34] Pretta A, Lai E, Persano M (2021). Uncovering key targets of success for immunotherapy in pancreatic cancer. Expert Opin. Ther. Targets.

[35] Donisi C, Puzzoni M, Ziranu P (2020). Immune checkpoint inhibitors in the treatment of HCC. Front Oncol..

[36] Lai E, Astara G, Ziranu P (2021). Introducing immunotherapy for advanced hepatocellular carcinoma patients: Too early or too fast?. Crit. Rev. Oncol. Hematol..

[37] Pardoll DM (2012). The blockade of immune checkpoints in cancer immunotherapy. Nat. Rev. Cancer..

[38] Casak SJ, Marcus L, Fashoyin-Aje L (2021). FDA approval summary: Pembrolizumab for the first-line treatment of patients with MSI-H/dMMR advanced unresectable or metastatic colorectal carcinoma. Clin. Cancer Res. Off. J. Am. Assoc. Cancer Res..

[39] Marcus L, Lemery SJ, Keegan P, Pazdur R (2019). FDA approval summary: Pembrolizumab for the treatment of microsatellite instability-high solid tumors. Clin. Cancer Res. Off. J. Am. Assoc. Cancer Res..

[40] Trullas A, Delgado J, Genazzani A (2021). The EMA assessment of pembrolizumab as monotherapy for the first-line treatment of adult patients with metastatic microsatellite instability-high or mismatch repair deficient colorectal cancer. ESMO Open..

[41] Lipson EJ, Sharfman WH, Drake CG (2013). Durable cancer regression off-treatment and effective reinduction therapy with an anti-PD-1 antibody. Clin. Cancer Res. Off. J. Am. Assoc. Cancer Res..

[42] Diaz LA, Le DT, Kim TW (2020). Pembrolizumab monotherapy for patients with advanced MSI-H colorectal cancer: Longer-term follow-up of the phase II, KEYNOTE-164 study. J. Clin. Oncol..

[43] O’Neil BH, Wallmark JM, Lorente D (2017). Safety and antitumor activity of the anti-PD-1 antibody pembrolizumab in patients with advanced colorectal carcinoma. PLoS ONE.

[44] Overman MJ, Lonardi S, Wong KYM (2018). Durable clinical benefit with nivolumab plus ipilimumab in DNA mismatch repair-deficient/microsatellite instability-high metastatic colorectal cancer. J. Clin. Oncol. Off. J. Am. Soc. Clin. Oncol..

[45] Lenz HJ, Lonardi S, Zagonel V (2021). Subgroup analyses of patients (pts) with microsatellite instability-high/mismatch repair-deficient (MSI-H/dMMR) metastatic colorectal cancer (mCRC) treated with nivolumab (NIVO) plus low-dose ipilimumab (IPI) as first-line (1L) therapy: Two-year clinical update. J. Clin. Oncol..

[46] André T, Lonardi S, Wong K (2021). SO-27 Nivolumab plus low-dose ipilimumab in previously treated patients with microsatellite instability-high/mismatch repair-deficient metastatic colorectal cancer: 4-year follow-up from CheckMate 142. Ann. Oncol..

[47] Lenz HJ, Van Cutsem E, Luisa Limon M (2022). First-line nivolumab plus low-dose ipilimumab for microsatellite instability-high/mismatch repair-deficient metastatic colorectal cancer: The phase II CheckMate 142 study. J. Clin. Oncol. Off. J. Am. Soc. Clin. Oncol..

[48] Andre T, Shiu KK, Kim TW (2021). Final overall survival for the phase III KN177 study: Pembrolizumab versus chemotherapy in microsatellite instability-high/mismatch repair deficient (MSI-H/dMMR) metastatic colorectal cancer (mCRC). J. Clin. Oncol..

[49] Fucà G, Cohen R, Lonardi S (2022). Ascites and resistance to immune checkpoint inhibition in dMMR/MSI-H metastatic colorectal and gastric cancers. J. Immunother. Cancer.

[50] Fabrizio DA, George TJJ, Dunne RF (2018). Beyond microsatellite testing: Assessment of tumor mutational burden identifies subsets of colorectal cancer who may respond to immune checkpoint inhibition. J. Gastrointest. Oncol..

[51] Schrock AB, Ouyang C, Sandhu J (2019). Tumor mutational burden is predictive of response to immune checkpoint inhibitors in MSI-high metastatic colorectal cancer. Ann. Oncol. Off. J. Eur. Soc. Med. Oncol..

[52] Bielska AA, Chatila WK, Walch H (2021). Tumor mutational burden and mismatch repair deficiency discordance as a mechanism of immunotherapy resistance. J. Natl. Compr. Cancer Netw. JNCCN.

[53] Domingo E, Freeman-Mills L, Rayner E (2016). Somatic POLE proofreading domain mutation, immune response, and prognosis in colorectal cancer: A retrospective, pooled biomarker study. Lancet Gastroenterol. Hepatol..

[54] Hu H, Cai W, Wu D (2021). Ultra-mutated colorectal cancer patients with POLE driver mutations exhibit distinct clinical patterns. Cancer Med..

[55] Cocco E, Benhamida J, Middha S (2019). Colorectal carcinomas containing hypermethylated MLH1 promoter and wild-type BRAF/KRAS are enriched for targetable kinase fusions. Cancer Res..

[56] Vaňková B, Vaněček T, Ptáková N (2020). Targeted next generation sequencing of MLH1-deficient, MLH1 promoter hypermethylated, and BRAF/RAS-wild-type colorectal adenocarcinomas is effective in detecting tumors with actionable oncogenic gene fusions. Genes Chromosomes Cancer..

[57] Hamada T, Zhang X, Mima K (2018). Fusobacterium nucleatum in colorectal cancer relates to immune response differentially by tumor microsatellite instability status. Cancer Immunol. Res..

[58] Gao Y, Bi D, Xie R (2021). Fusobacterium nucleatum enhances the efficacy of PD-L1 blockade in colorectal cancer. Signal Transduct. Target Ther..

[59] Chida K, Kawazoe A, Suzuki T (2022). Transcriptomic profiling of MSI-H/dMMR gastrointestinal tumors to identify determinants of responsiveness to anti-PD-1 therapy. Clin. Cancer Res. Off. J. Am. Assoc. Cancer Res..

[60] Sade-Feldman M, Jiao YJ, Chen JH (2017). Resistance to checkpoint blockade therapy through inactivation of antigen presentation. Nat. Commun..

[61] Lu J, Chatterjee M, Schmid H, Beck S, Gawaz M (2016). CXCL14 as an emerging immune and inflammatory modulator. J. Inflamm. Lond. Engl..

[62] Westrich JA, Vermeer DW, Silva A (2019). CXCL14 suppresses human papillomavirus-associated head and neck cancer through antigen-specific CD8(+) T-cell responses by upregulating MHC-I expression. Oncogene.

[63] Vivier E (2011). Innate or adaptive immunity? The example of natural killer cells. Science.

[64] Lin M (2017). Clinical efficacy of percutaneous cryoablation combined with allogenic NK cell immunotherapy for advanced non-small cell lung cancer. Immunol. Res..

[65] Okazaki T, Chikuma S, Iwai Y, Fagarasan S, Honjo T (2013). A rheostat for immune responses: The unique properties of PD-1 and their advantages for clinical application. Nat. Immunol..

[66] Kamata T (2016). Blockade of programmed death-1/programmed death ligand pathway enhances the antitumor immunity of human invariant natural killer T cells. Cancer Immunol. Immunother..

[67] Hsu J, Hodgins JJ, Marathe M (2018). Contribution of NK cells to immunotherapy mediated by PD-1/PD-L1 blockade. J. Clin. Invest..

[68] Dong W (2019). The mechanism of anti-PD-L1 antibody efficacy against PD-L1-negative tumors identifies NK cells expressing PD-L1 as a cytolytic effector. Cancer Discov..

[69] Concha-Benavente F, Kansy B, Moskovitz J, Moy J, Chandran U, Ferris RL (2018). PD-L1 mediates dysfunction in activated PD-1^+^ NK cells in head and neck cancer patients. Cancer Immunol. Res..

[70] Zeng J, Yang X, Cheng L (2013). Chemokine CXCL14 is associated with prognosis in patients with colorectal carcinoma after curative resection. J. Transl. Med..

[71] Platet N, Hinkel I, Richert L (2017). The tumor suppressor CDX2 opposes pro-metastatic biomechanical modifications of colon cancer cells through organization of the actin cytoskeleton. Cancer Lett..

[72] Björkman K, Jalkanen S, Salmi M (2021). A prognostic model for colorectal cancer based on CEA and a 48-multiplex serum biomarker panel. Sci. Rep..

[73] Scartozzi M, Giampieri R, Maccaroni E (2012). Pre-treatment lactate dehydrogenase levels as predictor of efficacy of first-line bevacizumab-based therapy in metastatic colorectal cancer patients. Br. J. Cancer.

[74] Mazaki J, Katsumata K, Kasahara K (2020). Neutrophil-to-lymphocyte ratio is a prognostic factor for colon cancer: A propensity score analysis. BMC Cancer.

[75] Rumpold H, Niedersüß-Beke D, Heiler C (2020). Prediction of mortality in metastatic colorectal cancer in a real-life population: A multicenter explorative analysis. BMC Cancer.

[76] Zeineddine FA, Zeineddine MA, Yousef A (2023). Survival improvement for patients with metastatic colorectal cancer over twenty years. npj Precis. Onc..

[77] Demurtas L, Puzzoni M, Giampieri R (2017). The role of primary tumour sidedness, EGFR gene copy number and EGFR promoter methylation in RAS/BRAF wild-type colorectal cancer patients receiving irinotecan/cetuximab. Br. J. Cancer.

[78] Hou W, Yi C, Zhu H (2022). Predictive biomarkers of colon cancer immunotherapy: Present and future. Front. Immunol..

